# Supplementary material for: Inequality in genetic cancer risk suggests bad genes rather than bad luck
Source: Nat Commun. 2017 Oct 27;8:1165. doi: 10.1038/s41467-017-01284-y (PMC5660094; doi:10.1038/s41467-017-01284-y)
Supplement: Supplementary file 2 — Description of Additional Supplementary Files [file 41467_2017_1284_MOESM2_ESM.pdf]

Description of Additional Supplementary Files

File Name: Supplementary Data 1
Description: In the Supplementary Data file, we have included the R code for all calculations as a txt file, that is in text format.
